# A review of respirable fine particulate matter (PM_2.5_)-induced brain damage

**DOI:** 10.3389/fnmol.2022.967174

**Published:** 2022-09-07

**Authors:** Wei Li, Guohui Lin, Zaixing Xiao, Yichuan Zhang, Bin Li, Yu Zhou, Yong Ma, Erqing Chai

**Affiliations:** ^1^The First Clinical Medical College of Gansu University of Chinese Medical, Lan Zhou, China; ^2^Cerebrovascular Disease Center of Gansu Provincial People's Hospital, Lan Zhou, China; ^3^Key Laboratory of Cerebrovascular Diseases in Gansu Province, Lan Zhou, China; ^4^Day Treatment Center II of Gansu Provincial Maternity and Child-Care Hospital, Lan Zhou, China; ^5^The First School of Clinical Medicine of Lanzhou University, Lan Zhou, China

**Keywords:** respirable fine particulate matter (PM_2.5_), brain injury, mechanism, review, self-repair of brain injury

## Abstract

Respirable fine particulate matter (PM_2.5_) has been one of the most widely publicized indicators of pollution in recent years. Epidemiological studies have established a strong association between PM_2.5_, lung disease, and cardiovascular disease. Recent studies have shown that PM_2.5_ is also strongly associated with brain damage, mainly cerebrovascular damage (stroke) and neurological damage to the brain (changes in cognitive function, dementia, psychiatric disorders, etc.). PM_2.5_ can pass through the lung–gas–blood barrier and the “gut–microbial–brain” axis to cause systemic oxidative stress and inflammation, or directly enter brain tissue *via* the olfactory nerve, eventually damaging the cerebral blood vessels and brain nerves. It is worth mentioning that there is a time window for PM_2.5_-induced brain damage to repair itself. However, the exact pathophysiological mechanisms of brain injury and brain repair are not yet fully understood. This article collects and discusses the mechanisms of PM_2.5_-induced brain injury and self-repair after injury, which may provide new ideas for the prevention and treatment of cerebrovascular and cerebral neurological diseases.

## Introduction

In recent years, with the expansion of urban scale and the increasingly serious air pollution, more and more attention has been paid to the impact of PM_2.5_ on human health. According to the Global Burden of Disease Study in 2017, about 4.9 million people died from air pollution worldwide, and respirable fine particulate matter (PM_2.5_) accounted for about 60% of deaths (GBD 2017 Risk Factor Collaborators, 2018), making air pollution a global problem affecting human health. The polluted air is a mixture rich in harmful components, such as respirable fine particles, carbon monoxide, lead, nitrogen dioxide, ozone, and sulfur dioxide, of which respirable fine particles (PM_2.5_) are one of the most concerning environmental pollution indicators. PM_2.5_ is particulate matter with a kinetic equivalent diameter of less than or equal to 2.5 microns in the atmosphere. It is rich in a large amount of toxic and harmful substances and has the characteristics of long residence time in the atmosphere and long transportation distance. It has a significant influence on human health and air quality (Brook et al., 2010). The main sources of PM_2.5_ are natural and anthropogenic sources, with anthropogenic sources accounting for the major part (Kim et al., 2015). Numerous studies have shown that PM_2.5_ can cause not only respiratory diseases (allergic airway inflammation, asthma, and chronic obstructive pulmonary disease) (Choi et al., 2018; Weinmayr et al., 2018), but also diseases outside the respiratory system, such as cardiovascular disease, diabetes, childhood obesity, and cancer (Desikan, 2017; Mao et al., 2017; Maji et al., 2018; Wu et al., 2021).

PM_2.5_ enters the lung tissue at the end of the respiratory tract through the central airway after being filtered by nasal hairs, and the accumulated PM_2.5_ stimulates oxidative stress and inflammation in the lung, and a large amount of inflammatory factors enter the blood through the air–blood barrier and cause systemic inflammation and brain tissue damage (Costa et al., 2017). In addition, PM_2.5_ can also directly cross the olfactory nerve and cause damage to the blood–brain barrier (Oberdörster et al., 2004), or PM_2.5_ can cause dysbiosis of the intestinal flora and cause brain damage through the “gut–brain axis” (Shou et al., 2019). The changes in the brain after PM_2.5_ exposure mainly lie in the damage to cerebral blood vessels, the damage of cranial nerves, and the formation of brain tumors. The cerebrovascular damage is mainly in terms of stroke, while the neurological damage is mainly in terms of altered cognitive function, dementia, and psychiatric disorders (Kioumourtzoglou et al., 2016; Qiu et al., 2017). PM_2.5_ has been demonstrated to be a potentially important variable risk factor for stroke, especially in low- and middle-income countries (Feigin et al., 2016). The psychiatric and neurological symptoms caused by neurological damage to the brain often lead to disability, reduce the number of years of survival, largely affect the quality of life and workforce, and increase the burden on families. PM_2.5_-induced neurological brain injury has been extensively studied in recent years. Kioumourtzoglou et al. (2016) conducted a large-scale, multi-site epidemiological study in the northeastern U.S. and found that cities with long-term PM_2.5_ exposure increased the risk of Alzheimer's disease and Parkinson's disease in local residents. In addition, PM_2.5_ exposure may also increase the risk of brain tissue atrophy and aging and lead to damage to brain white matter. Although this study has various limitations, such as region and ethnicity, it still provides a basis for further exploration of large epidemiological studies. PM_2.5_ is widely present in the air, and a comprehensive understanding of the relationship between PM_2.5_ and brain injury may provide new ideas for the prevention and treatment of brain injury, and a review of the relationship between PM_2.5_ and brain injury and the pathogenesis is necessary to discuss.

## PM_2.5_ exposure-induced brain outcomes

### Cerebrovascular damage

Currently, the research on cerebral vascular damage induced by PM_2.5_ is mainly manifested in vascular endothelial cell dysfunction, intracranial atherosclerosis, and stroke (ischemic stroke and hemorrhagic stroke). Long et al. studied the effect of PM_2.5_ on vascular endothelial function in ApoE-/- mice exposed to PM_2.5_ many times. The results showed that NO is the initial factor of oxidative stress induced by PM_2.5_ exposure, and oxidative stress leads to inflammation and vascular dysfunction (Long et al., 2020). In addition, Hu et al. (2021) found through *in vivo* and *in vitro* experiments that PM_2.5_ exposure leads to a significant relationship between NLRP3 inflammasome activation and vascular endothelial dysfunction. Vascular endothelial dysfunction is the initial pathological change of atherosclerosis, and PM_2.5_ aggravating intracranial atherosclerosis has been confirmed in animal experiments. A study by Guan et al. found that exposure of adult Sprague-Dawley rats to PM_2.5_ for 12 weeks significantly aggravated intracranial atherosclerosis (Guan et al., 2017). O'Donnell et al. (2011) through the analysis of 9,202 hospitalized patients with acute ischemic stroke, it was found that the correlation between PM_2.5_ and the risk of ischemic stroke varied with different causes of stroke, and the correlation between stroke caused by large artery atherosclerosis was the strongest. A meta-analysis of 20 relevant studies by Scheers et al. (2015) found that for each 5-ug/m^3^ increase in PM_2.5_ concentration in Europe and North America, the hazard ratios for incident stroke and stroke mortality were 1.064 (1.021–1.109) and 1.125 (1.007 −1.256). A study by Leiva et al. (2013) found an association between PM_2.5_ exposure and stroke admissions, with each 10-ug/m^3^ increase in PM_2.5_ concentration associated with a 1.29% increase in the risk of emergency admission for cerebrovascular disease. Zhang R. et al. (2018) found that elevated PM_2.5_ levels were associated with increased ischemic and hemorrhagic stroke mortality [0.23% (95% CI, 0.04–0.42%), 0.37% (95% CI, 0.07–0.67%)], whereas elevated PM_10_ levels were only associated with ischemic stroke mortality. Yang et al. (2021) further demonstrated that PM_2.5_ should be considered an additional risk factor for ischemic stroke by conducting a meta-analysis of the literature on the relationship between PM_2.5_ and ischemic stroke in Taiwan, China, and that short-, medium-, and long-term exposure to ambient PM_2.5_ increases the risk of ischemic stroke. Compared with ischemic stroke, the findings of PM_2.5_ associated with hemorrhagic stroke are inconsistent across countries. Most studies in Western countries have shown that PM_2.5_ is significantly associated with ischemic stroke, but not with hemorrhagic stroke; while studies in Asian countries have shown an association between PM_2.5_ and hemorrhagic stroke. For example, a Japanese study also reported a stronger effect of PM pollution on hemorrhagic stroke [1.041 (95% confidence interval: 1.011–1.072)] than on ischemic stroke (Yorifuji et al., 2011), while studies in the United States and European countries have shown that PM pollution is associated with an ischemic stroke rather than hemorrhagic stroke (Wellenius et al., 2015). In conclusion, PM_2.5_ is closely related to cerebrovascular disease, and controlling air pollution may be a way to reduce cerebrovascular morbidity and mortality.

### Brain nerve damage

The damage of PM_2.5_-induced cranial nerves is mainly manifested in neurological and psychiatric diseases caused by neurotoxic effects, such as Alzheimer's disease, Parkinson's disease, cognitive impairment, depression, anxiety, and autism. There are currently studies in animal experiments and clinical trials. Ku et al. (2017) conducted a study using the Morris water maze to test the spatial learning and memory ability of C57BL/6 mice after inhaling PM_2.5_ for 4 weeks. The results showed that the spatial learning and memory ability of mice decreased significantly after inhaling PM_2.5_. A study by Nephew et al. (2020) found that long-term exposure to traffic-related PM in rats and their offspring during pregnancy and lactation reduces social behavior, increases anxiety, impairs cognition, reduces levels of inflammation and growth factors (associated with behavioral changes), and undermines the neurointegrity of young male offspring. This further proves that PM is closely related to mental disorders. There is also experimental evidence that PM_2.5_ exposure can lead to early Alzheimer's disease (AD)-related pathology in transgenic AD mice, leading to AD-related molecular and cellular changes, such as mitochondrial dysfunction, synaptic defects, axonal growth impairment, nerve cell death, glial cell activation, neuroinflammation and neurovascular dysfunction, and increased levels of amyloid β (Aβ) and tau phosphorylation. These changes are the basis of cognitive impairment associated with AD (Wang L. et al., 2020). A 2016 study published in Environmental HealthPerspectives showed that long-term exposure to PM_2.5_ increases the risk of dementia, Alzheimer's disease, and Parkinson's disease in humans (Kioumourtzoglou et al., 2016). A large body of epidemiological evidence suggests that PM_2.5_ exposure in the elderly may lead to memory impairment (Fonken et al., 2011), and exposure to high levels of PM_2.5_ is strongly associated with increased risk of neuropathic dysfunction, cognitive decline, and accelerated brain aging (Power et al., 2011; Wellenius et al., 2015), and studies by Elder et al. (2006) have demonstrated that PM_2.5_ is a major cause of learning and memory impairment. Sunyer et al. (2015) recruited 2,715 children aged 7–10 years in 39 schools in Barcelona in a prospective study, which showed that children attending schools with high traffic pollution showed less cognitive improvement, which further demonstrates the toxic effect of PM_2.5_ in terms of brain neurological damage. The damage to the brain nerves seriously affects the quality of life of the organism, and since the number of neurodegenerative diseases is increasing year by year in recent years with the increase of global aging, it is necessary to carry out an in-depth study of PM_2.5_ as one of the risk molecules that induce brain nerve damage.

### Brain tumor formation

Epidemiological evidence on the relationship between PM_2.5_ and brain tumors is scarce, but based on PM_2.5_-induced neuroinflammation and oxidative stress, this may also contribute to brain tumor formation. Jørgensen et al. (2016) conducted a study on the relationship between long-term exposure to air pollution and the risk of brain tumors and found that the number of brain tumors was weakly positively correlated with PM_2.5_, NO_2_, and NOx. Meningeal tumors were more strongly associated with PM_2.5_ and NO_2_ than tumors located in the brain, and benign tumors were more strongly associated with malignant tumors. Weichenthal et al. (2020) conducted a cohort study on the spatial differences of environmental ultrafine particulate matter in 1.9 million Canadian adults and found that every 10,000/cm^3^ increase in the environmental ultrafine particulate matter was positively correlated with the incidence of brain tumors. Ultrafine particulate matter in the environment may be a risk factor for the development of brain tumors in adults. However, McKean-Cowdin et al. (2009) failed to find an association between brain tumor mortality (*n* = 1,284) and residential exposure to PM_2.5_ and PM10 or NO_2_ in a large US cancer prevention study cohort. Whether there is a correlation between PM_2.5_ and brain tumor formation is still controversial, and more research is needed to discover the link between PM_2.5_ and brain tumors in the future.

## Underlying mechanisms of brain injury

**Figure 1:**
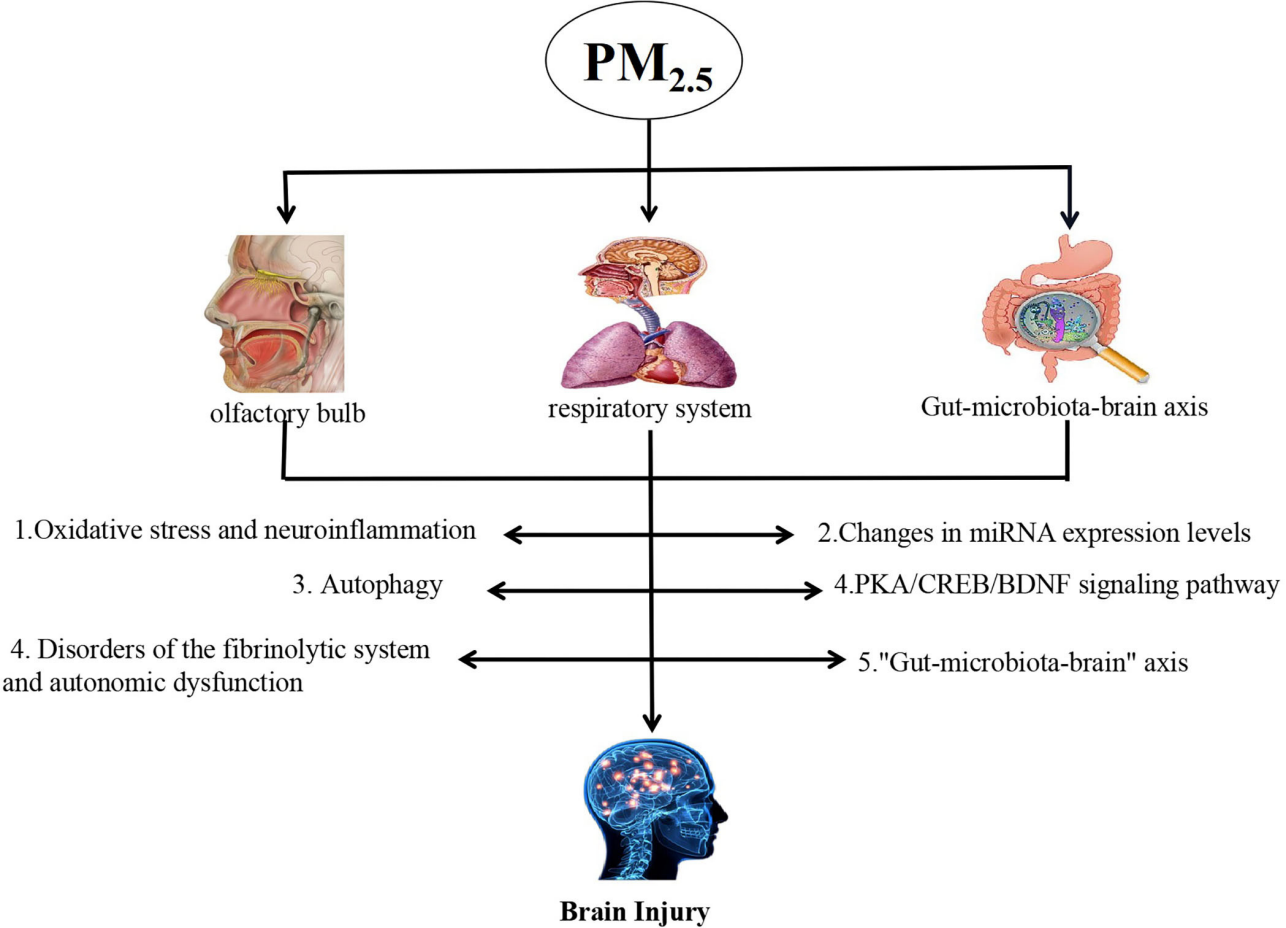
The pathways involved in PM_2.5_-induced brain damage.

PM_2.5_ can cause brain damage in a variety of ways, but the specific mechanism of inducing brain damage has not been fully clarified so far. Now, the existing research mechanism is discussed (shown in Figure 1).

### Oxidative stress and neuroinflammation

ROS-mediated MAPK signaling pathway PM_2.5_-induced oxidative stress is considered to be the key molecular mechanism of PM_2.5_-mediated toxicity. Oxidative stress is caused by an imbalance between reactive oxygen species (ROS) production and antioxidant mechanisms. Exposure of cells to ROS or ROS-generating systems can activate various cellular signaling pathways (Eckers and Klotz, 2009). MAPKs are a group of protein serine/threonine kinases that are activated in response to various extracellular stimuli and mediate signal transduction from the cell surface to the nucleus, including c-Jun NH2-terminal kinase (JNK), extracellular signal regulation Kinase (ERK), and p38 MAPK (Davis, 2000). ROS can lead to the continuous activation of p38 MAPK through various pathways, such as activation of MAPK kinase and inhibition of MAPK phosphatase (Talwar et al., 2017). Rui et al. (2016) found that PM_2.5_ induced phosphorylation of N-terminal kinase (JNK), extracellular signal-regulated kinase (ERK), p38 mitogen-activated protein kinase (MAPK), and protein kinase B (AKT) of Jun by exposing human umbilical vein cell line EA.hy926 to PM_2.5_, and activated nuclear factor kappa B (NF-κB). ROS may act as a signal molecule to trigger the expression of ICAM-1 and VCAM-1 by activating the ERK/AKT/NF-κB-dependent pathway, and further promoting the adhesion of monocytes to endothelial cells. PM_2.5_ exposure can also induce oxidative stress through activation of the MAPK/AP-1 cascade, leading to upregulation of angiotensin II type 1 receptor (AT1R) and vascular endothelial cell dysfunction (Xu et al., 2019).

PI3K/AKT/FoxO1 pathway PI3K/Akt is an important signaling pathway related to oxidative stress, and the upregulation of this pathway will lead to an increase in the production of reactive oxygen species in the body (Morimoto et al., 2013). FoxO1 is a member of the FoxO family, which is an important transcription factor that can regulate various physiological and pathological processes, such as oxidative stress, inflammatory response, energy metabolism, and apoptosis. However, AKT can inactivate FoxO1 phosphorylation, rendering its regulatory role ineffective. Song et al. (2021) found that long-term exposure to PM_2.5_ in rats increased the mRNA expression of PI3K and AKT in brain tissue, and decreased the mRNA expression of FoxO1. Brain injury induced by PM_2.5_ may be related to the activation of the PI3K/AKT/FoxO1 pathway.

Inflammatory genes can be activated under the drive of oxidative stress, producing a large number of inflammatory mediators (tumor necrosis factor-α, interleukin (IL)-1, IL-6, IL-17, and IL-10) involved in the systemic chronic inflammation reaction. Studies have found that PM_2.5_ can also activate the nuclear factor-κB signaling pathway, and then induce the high expression of inflammatory mediators, such as IL-6, IL-8, IL-1β, and tumor necrosis factor-α, causing extensive pulmonary inflammatory lesions (He et al., 2017; Wang Y. et al., 2020). These inflammatory mediators become the external stimulus transduction signals required for the activation of the nuclear factor-κB pathway, which further activates the nuclear factor-κB signaling pathway, induces an inflammatory cascade, and aggravates neuroinflammation (Zhang Y. et al., 2018). PM_2.5_ exposure can also activates the NLRP3 inflammasome, induces increased production of IL-1β and IL-18, causes systemic and neuroinflammation, and leads to endothelial and target organ damage (Gao et al., 2022).

### Changes in miRNA expression levels

MicroRNAs (miRNAs) are a group of non-coding RNA molecules of 18–25 nucleotides that are increasingly recognized as key regulators of proteins at the translational level (Bartel, 2009). It is also a potent regulator of nervous system development, function, and disease. Alterations in miRNA expression levels are closely associated with cognitive degeneration and neurodegenerative diseases, including AD (Pichler et al., 2017). A study (Chao et al., 2017) found that fetal mice exposed to PM_2.5_ increased the expression levels of miR-6315, miR-3588, and miR-466b-5p in the cerebral cortex. These genes were positively correlated with *PKn2* (astrocyte migration), *Gorab* (neuritogenesis), and *Mobp* (which induces experimental allergic encephalomyelitis). PM_2.5_ also decreased the expression of miR-338-5p and let-7e-5p, both genes involved in intellectual development. In addition, PM_2.5_ exposure decreased the expression of MiR99b-5p, miR-92b-5p, and miR-99a-5p in the hippocampus and inhibited the learning and motor coordination of fetal mice. β-Site amyloid precursor protein (APP) cleaving enzyme 1 (β-secretase, BACE1) is a key enzyme that catalyzes the production of amyloid β (A β) peptides from APP. The activation of BACE1 is a sign of early cognitive impairment and plays an important role in the gradual transformation into AD. Previous studies (Ku et al., 2017) have found that PM_2.5_ inhalation impairs synaptic and cognitive function mediated by NF-κB p65-induced downregulation of miR-574-5p against BACE1. Ma et al. (2022) found that the high expression of MicroRNA-29b (miR-29b) may regulate apoptosis and oxidative stress, which is an important factor leading to neuronal injury after cerebral ischemia.

### Autophagy

Autophagy is a general term for the degradation of intracellular substances through lysosomes after cells are stimulated. Moderate autophagy in the early stage of the body has a protective effect, while over-activated autophagy will aggravate the damage to the body's function. In the rat model of focal cerebral ischemia (MCAO), early autophagy has neuroprotective effects, while overactivated autophagy aggravates neurological deficits (Gao et al., 2012). Wang et al. (2021) found in rotenone-treated PC12 cells *in vivo* and *in vitro* studies on PM_2.5_ and neurodegenerative diseases, exposure to PM_2.5_ can reduce the LC3II/LC3I ratio and the expression level of Atg5, and increase the mammalian target of rapamycin (mTOR) expression levels, suggesting that PM_2.5_ exposure inhibited autophagy. Autophagy in the substantia nigra of Parkinson's mice following PM_2.5_ inhalation *in vitro* changes consistent with cellular models. However, Ren et al. (2021) found an increase in the level of Lc3 and a decrease in the level of p62 in brain tissue after PM_2.5_ exposure in evaluating the effect of PM_2.5_ on brain injury in mice, indicating that PM_2.5_ exposure increased the level of autophagy. Moreover, after exposure to PM_2.5_, the level of AMPK increased and the level of MTOR decreased, suggesting that PM_2.5_ may induce autophagy by activating the AMPK/mTOR pathway.

### PKA/CREB/BDNF signaling pathway

Protein kinase A (PKA) is an important regulator of various cellular processes. As the major upstream kinase of cAMP response element binding protein (CREB), PKA can phosphorylate CREB, thereby promoting the recruitment of transcription factor components to its promoter, including the brain-derived neurotrophic factor (BDNF) gene. PKA can promote the transcription of BDNF and expression, thereby exerting neuroprotective effects (Leal et al., 2017). The PKA/CREB/BDNF signaling pathway is responsible for promoting neuronal survival, regulating synaptic morphology, and enhancing synaptic transmission efficiency (Zhong et al., 2018). Liu et al. (2019) found that neonatal rats' early exposure to PM_2.5_ can lead to synaptic damage and emotional and cognitive impairment, possibly through the CREB/BDNF signaling pathway. Liu et al. (2021) found upregulation by PM_2.5_ in cultured hippocampal neurons (DIV3) or downregulation of the PKA/CREB/BDNF signaling pathway can alleviate or aggravate PM_2.5_-induced neuronal damage to varying degrees, further demonstrating that PKA/CREB/BDNF pathway may play an important role in PM_2.5_-mediated neurodevelopmental toxicity.

### Disorders of the fibrinolytic system and autonomic dysfunction

PM_2.5_ can lead to changes in coagulation and fibrinolytic system homeostasis. PM_2.5_ induces overexpression of tissue factor (TF), which activates the receptor and cofactor for coagulation factor VIIa (F VIIa), and the formation of the TF/FVIIa complex initiates the exogenous coagulation pathway and promotes blood hypercoagulation, which is thought to be a key pathway for thrombin production *in vivo*. Moreover, the inflammatory reaction mediated by PM_2.5_ can change the balance between tissue plasminogen activator and plasminogen activator inhibitor-1, resulting in the imbalance of fibrinolysis (Rückerl et al., 2014), which greatly increases the risk of thrombosis.

PM_2.5_ induces autonomic dysfunction, and this impairment appears to be attributable to stimulation of the HPA axis (hypothalamic–pituitary–adrenal axis) following hypothalamic inflammation induced by PM_2.5_ exposure in this setting. The activated central nervous system amplifies or modulates systemic cardiometabolic responses by stimulating the hypothalamic-pituitary-adrenal (HPA) axis, manifested as corticotropin-releasing hormone (CRH), adrenocorticotropic hormone (ACTH), and corticotropin Increased secretion of alcohol (Li et al., 2017). Ultimately, it disrupts cardiovascular and cerebrovascular homeostasis, damages vascular function, affects hemodynamics, and increases blood pressure.

### “Gut–microbiota–brain” axis

The gut microbiota is the normal microbiota present in the gut. It is closely linked to human metabolism, gut homeostasis, immune development (Lynch and Pedersen, 2016), and brain development processes and behavior (Mayer et al., 2015; Morais et al., 2021). A stable and diverse intestinal flora is optimal for maintaining health. Alterations or dysbiosis of the gut microbiota can trigger a variety of diseases, such as inflammatory bowel disease (Fasano, 2020), celiac disease (Odenwald and Turner, 2017), metabolic syndrome (Fan and Pedersen, 2021), diabetes (Sorini et al., 2019), colon cancer (Fidelle et al., 2020), autism, anxiety, depression, and neurodegenerative diseases (Rutsch et al., 2020; Zhu et al., 2020). The link between gut flora and the physiological functions of the brain is described as the “gut–microbe–brain” axis (Lombardi et al., 2018). It has been shown that disruption or interruption of any of the communication pathways between these microbes and the brain may trigger an inflammatory response in the organism. Pathogenic microbes release metabolites and molecules that trigger cytokines in the host and cause inflammation in the central nervous system, greatly contributing to the development and progression of brain disease (Zhu et al., 2020). PM_2.5_ can enter the gastrointestinal tract either directly or indirectly. Some studies have shown that exposure to PM through inhalation can alter the composition of the gut microbiota across the gastrointestinal tract, increasing the permeability of the intestinal barrier and increasing the chances of pathogens, such as bacteria, crossing the intestinal mucosa and entering the circulatory system (Mutlu et al., 2018). PM_2.5_ exposure can induce the secretion of inflammatory cytokines from intestinal epithelial cells through the damaged intestinal barrier from the peripheral system to the central nervous system, leading to neuroinflammation. Recent evidence has demonstrated a relationship between microbiome changes and the pathophysiology of AD (Köhler et al., 2016) and an association between microbial populations and the development of Alzheimer's disease has been found (Vogt et al., 2017).

## Self-repair after brain injury

Self-repair after PM_2.5_-induced brain injury is also an important issue of concern. Previous studies have found that after continuous exposure of C57BL/6 male mice to high levels of PM_2.5_ for 4, 8, and 12 weeks, the mice exhibited an injury–repair–imbalance response that ultimately led to depressive behavior and upregulation of pro-inflammatory cytokines (Liu et al., 2018). Ji et al. (2019), in a study of the effects and subsequent recovery at the end of PM_2.5_ exposure, found that inflammatory cytokines and chemokines were persistently elevated in mice after 4 weeks of PM_2.5_ exposure, but these effects all returned to normal levels within 2 weeks after cessation of exposure. A study by Ren et al. (2020) also confirmed that PM_2.5_-induced damage to the organism could repair itself after cessation of exposure, but their further study found that this type of repair could not be considered a true repair. This is because significant changes were observed between groups when re-exposed to the same dose of PM_2.5_, which instead increased the susceptibility of target organs. Song et al. (2021) also found in a study on the mechanisms of PM_2.5_-induced brain damage that there may be antioxidant, anti-inflammatory, fibrinolytic, and other partial physiological processes in the organism during 2–4 months of PM_2.5_ exposure in rats, but this finding needs to be further investigated. Therefore, it is necessary to evaluate PM_2.5_-induced injury with increasing exposure time, which can help to identify early biological indicators and clarify potential time points of repair or deterioration in the injury process, which will provide important information in the prevention of PM_2.5_-induced brain injury in the future.

## Conclusion

Nowadays, as the problem of air pollution around the world intensifies, exposure to PM_2.5_ in air pollution has also become a problem that threatens human health and safety. The results of animal experiments and epidemiological experiments at home and abroad have shown that exposure to PM_2.5_ can cause serious respiratory and cardiovascular diseases, as well as damage to the nervous system in many ways. The existing damage mechanisms mainly include oxidative stress and neuroinflammatory pathways, changes in miRNA expression levels, autophagy, PKA/CREB/BDNF signaling pathways, and disorders of the HPA axis. The “gut-microbe-brain” axis is also a research hotspot in recent years. The damage after exposure to PM_2.5_ is not a single mechanism, it is a chain reaction. The body's self-repair after PM_2.5_-induced brain injury is also worthy of further study. Identifying potential repair or deterioration time points in the process of injury and early prevention and control can greatly reduce the degree of brain damage. However, due to the complexity and diversity of PM components in the environment and the unclear toxicity, it brings challenges to the research of PM_2.5_-induced brain injury. Further clarification of the induced brain injury and the unresearched mechanism is our future efforts.

## Author contributions

WL participated in the design of the study, collected and analyzed the literature, and drafted and revised the manuscript. GL, ZX, YZha, BL, YZho, and YM analyzed the literature. EC designed the study and revised the manuscript. All authors contributed to the article and approved the submitted version.

## Conflict of interest

The authors declare that the research was conducted in the absence of any commercial or financial relationships that could be construed as a potential conflict of interest.

## Publisher's note

All claims expressed in this article are solely those of the authors and do not necessarily represent those of their affiliated organizations, or those of the publisher, the editors and the reviewers. Any product that may be evaluated in this article, or claim that may be made by its manufacturer, is not guaranteed or endorsed by the publisher.
